# Effectiveness and Safety of Rivaroxaban Versus Warfarin among Nonvalvular Atrial Fibrillation Patients with Concomitant Obstructive Sleep Apnea

**DOI:** 10.1055/a-2013-3346

**Published:** 2023-03-29

**Authors:** Nitesh Sood, Veronica Ashton, Youssef Bessada, Katelyn Galli, Brahim K. Bookhart, Craig I. Coleman

**Affiliations:** 1 Arrhythmia Services, Southcoast Health, Massachusetts, United States; 2 Real World Value and Evidence, Janssen Scientific Affairs LLC, Titusville, New Jersey, United States; 3 University of Connecticut School of Pharmacy, Connecticut, United States; 4 Evidence-Based Practice Center, Hartford Hospital, Hartford, Connecticut, United States

**Keywords:** rivaroxaban, warfarin, sleep Apnea, obstructive, atrial fibrillation, observational studies

## Abstract

**Background** Obstructive sleep apnea (OSA) is associated with an increased incidence of atrial fibrillation (AF), hypertension, diabetes, heart failure, coronary heart disease, stroke, and death. We sought to evaluate the effectiveness and safety of rivaroxaban versus warfarin in nonvalvular AF (NVAF) patients with concomitant OSA.

**Methods** This was an analysis of electronic health record (EHR) data from November 2010 to December 2021. We included adults with NVAF and OSA at baseline, newly initiated on rivaroxaban or warfarin, and with ≥12 months of prior EHR activity. Patients with valvular disease, alternative indications for oral anticoagulation, or who were pregnant were excluded. The incidence rates of developing stroke or systemic embolism (SSE) and bleeding-related hospitalization were evaluated. Hazard ratios (HRs) and 95% confidence intervals (CIs) were calculated using propensity score-overlap weighted proportional hazards regression. Multiple sensitivity and subgroup analyses were performed.

**Results** We included 21,940 rivaroxaban (20.1% at the 15 mg dose) and 38,213 warfarin (time-in-therapeutic range = 47.3 ± 28.3%) patients. Rivaroxaban was found to have similar hazard of SSE compared to warfarin (HR = 0.92, 95% CI = 0.82–1.03). Rivaroxaban was associated with a reduced rate of bleeding-related hospitalizations (HR = 0.85, 95% CI = 0.78–0.92) versus warfarin, as well as reductions in intracranial (HR = 0.76, 95% CI = 0.62–0.94) and extracranial (HR = 0.89, 95%CI = 0.81–0.97) bleeding. Upon sensitivity analysis restricting the population to men with a CHA _2_ DS _2_ VASc score ≥2 or women with a score ≥3, rivaroxaban was associated with a significant 33% risk reduction in SSE and 43% reduction in the risk of bleeding-related hospitalization. No significant interaction for the SSE or bleeding-related hospitalization outcomes was observed upon subgroup analyses.

**Conclusion** Among patients with NVAF and OSA, rivaroxaban had similar SSE risk versus warfarin but was associated with reductions in any intracranial and extracranial bleeding-related hospitalizations. Rivaroxaban was associated with significant reductions in SSE and bleeding-related hospitalizations when the study population was restricted to patients with a moderate-to-high risk of SSE. These data should provide prescribers with additional confidence in selecting rivaroxaban in NVAF patients who have OSA at the time of anticoagulation initiation.

## Introduction

Atrial fibrillation (AF) is the most common sustained supraventricular arrhythmia encountered in clinical practice in both the United States and worldwide. 1 Compared to the general population, AF increases patients' risk of stroke by approximately fivefold, as well as their risk of morbidity and mortality. 1

Obstructive sleep apnea (OSA) is characterized by recurrent episodes of partial (obstructive hypopnea) or complete obstruction (obstructive apnea) of the upper airway leading to reduced or absent breathing during sleep. 2 Continued nightly intermittent airway obstruction has been demonstrated to result in large swings in negative intrathoracic airway pressure, intermittent hypoxia, repeated arousals from sleep and neurohumoral activation, each of which contributes to an increased risk of adverse cardiovascular events. 3 OSA has been shown to be associated with an increased incidence of AF, hypertension, diabetes, heart failure, coronary heart disease, stroke, and death. 2 4

OSA and AF frequently coexist, and the relationship between them is likely bidirectional. 4 5 Data suggest that 21 to 74% of patients with AF are thought to have concomitant OSA. 4 OSA has been shown to reduce the efficacy of catheter-based 6 and pharmacological antiarrhythmic management. 7 In some, but not all studies, OSA has been shown to be an independent predictor of stroke in patients with AF. 8 9 10 11

Oral anticoagulation (OAC) with either a direct-acting oral anticoagulant (DOAC) or vitamin K antagonist (VKA) significantly decreases the risk of cardioembolic stroke in AF patients. 5 DOACs, including rivaroxaban, are recommended as first-line oral anticoagulants in the management nonvalvular AF (NVAF). 5 Factor Xa inhibition by rivaroxaban has been shown to prevent oxidative stress and fibrosis due to OSA-induced intermittent hypoxia, which could lead to reduced cardiovascular events. 12 To date, no study has assessed the effectiveness and safety of rivaroxaban compared to VKA therapy in patients with NVAF and OSA.

In the present study, we sought to evaluate the effectiveness (stroke or systemic embolism) and safety (bleeding-related hospitalization) of rivaroxaban versus warfarin in NVAF patients with OSA in routine clinical practice.

## Methods

We performed a cohort analysis within the US Optum De-Identified EHR data set. 13 EHR data from November 1, 2010 through December 31, 2021 were utilized for this study. Rivaroxaban was approved for NVAF in the United States in November 2011, and therefore, utilization of data back to November 2010 was required to provide a full 12-month preindex period for all patients. The EHR data set includes longitudinal patient-level medical record data for 91+ million patients seen at 700+ hospitals and 7,000+ clinics across the United States. This database contains data on insured and uninsured patients of all ages to provide a representative sample of United States patients with NVAF. It includes records of prescriptions and over-the-counter medications (as prescribed or self-reported by patients), laboratory results, vital signs, anthropometrics, other clinical observations, diagnoses (International Classification of Diseases [ICD-9] and ICD-10), and procedures codes (ICD-9, ICD-10, Current Procedural Terminology-4, Healthcare Common Procedure Coding System, Revenue codes). The use of the provided data set was determined by the New England Institutional Review Board to not constitute research involving human subjects and was therefore exempt from board oversight.

### Cohort Selection

Adult patients (≥18 years of age) with NVAF and comorbid OSA diagnosed during the baseline period including the index date, who were OAC naive, newly initiated on rivaroxaban or warfarin after November 1, 2011 (defined as the index date), active in the data set for ≥12 months prior to the index date and with documented care in the EHR from ≥1 provider in the 12 months prior to the index date were eligible for study inclusion. Patients with valvular heart disease (defined as any rheumatic heart disease, mitral stenosis, mitral valve repair, or replacement), any prior OAC use per written/electronic prescription or patient self-report during the 12-month preindex period, known to have received rivaroxaban doses other than 15 mg or 20 mg once daily, having venous thromboembolism as an alternative indication for OAC use, having undergone recent orthopedic knee or hip replacement within the prior 35 days, or who were pregnant were excluded.

The identification of OSA was based upon the presence of an ICD-9 and/or -10 billing code 14 of 327.20 (organic sleep apnea, unspecified), 327.23 (OSA [adult, pediatric]), 327.29 (other organic sleep apnea), 780.51 (insomnia with sleep apnea), 780.53 (hypersomnia with sleep apnea), 780.57 (sleep apnea [NOS]), G47.30 (sleep apnea, unspecified), G47.33 (OSA [adult, pediatric]), and G47.39 (other sleep apnea) during the 12-month baseline period. While select codes may have included central sleep apnea, we felt it likely that in most cases, such codes were associated with predominantly OSA and that it is important not to overly restrict case selection by avoiding codes that do not differentiate between central and obstructive. This coding schema has been shown to have a positive predictive value of >90% for the identification of OSA, 14 with additional data suggesting that PPV improves in the presence of comorbidities frequently present in AF patients (e.g., hypertension and diabetes). 15

### Confounder Adjustment and Handling of Missing Data

To adjust for potential confounding between the rivaroxaban and warfarin cohorts, we calculated propensity scores using multivariable logistic regression. 16 The multivariable logistic regression model included all covariates included in the baseline characteristics table. The presence of comorbid disease diagnoses was determined based upon billing codes and/or supporting laboratory and observation data. The absence of data suggesting a comorbidity exists was assumed to represent the absence of the disease. Consequently, all categorical covariates had complete data for all patients. When dependence on billing codes was required to identify covariates, we utilized validated or endorsed coding algorithms, whenever possible. 17 18 19 20 For continuous laboratory and observation variables with <25% values missing, data were imputed using multiple imputations based upon a fully conditional specification linear regression model, with all other available covariates and the outcomes included in the model. 21 Generated propensity scores were then used to weight patients for analysis using overlap weighting as described by Thomas and colleagues. 22 This approach assigns weights to patients that are proportional to their probability of belonging to the alternative treatment cohort. Rivaroxaban patients were weighted by the probability of receiving warfarin (i.e., 1—the propensity score), and warfarin patients were weighted by the probability of receiving rivaroxaban (i.e., propensity score).

Overlap weighting was utilized for confounder adjustment because it allows for all eligible patients to be included in the analysis, it assigns the greatest weight to patients in which treatment cannot be predicted (and the least weight to patients with extreme propensity scores), and because overlap weighting has the favorable property of exactly balancing all variables included in the multivariable logistic regression model used to derive the propensity score, resulting in absolute standardized differences (ASD) = 0 for each covariate.

### Outcomes

Our primary effectiveness outcome was stroke or systemic embolism (SSE) which included ischemic stroke, systemic embolism, or intracranial hemorrhage (ICH) identified using ICD-10 codes I60-I62 and I74 (and corresponding ICD-9 codes per Centers for Medicare and Medicaid Services General Equivalence Mapping files). 23 The primary safety outcome was bleeding-related hospitalization based on the validated Cunningham algorithm. 23 24 Secondary outcomes included ischemic stroke, ICH, and extracranial bleeding as separate outcomes.

### Statistical Analysis

Baseline characteristics were analyzed using descriptive statistics. Categorical variables were reported as proportions and continuous variables as means ± standard deviations. Propensity score-overlap weighted Cox proportional hazards regression models including index anticoagulation cohort (rivaroxaban or warfarin) as the only covariate and implementing a robust sandwich estimator were utilized to calculate hazard ratios (HRs) and 95% confidence intervals (CIs). The proportional hazard assumption was tested based on Schoenfeld residuals (and was found valid in all cases). Patients were followed until outcome occurrence, end-of-EHR activity, or end-of-data availability (intent-to-treat approach).

A sensitivity analysis in which stabilized inverse probability of treatment weighting (sIPTW) was utilized instead of OLW was performed. We also performed a sensitivity analysis in which we restricted the study population to patients at moderate-to-high risk of SSE (CHA _2_ DS _2_ VASc ≥2 for men, ≥3 for women). Subgroup analyses stratifying patients by age (≥75 years or <75 years), sex, obesity (body mass index [BMI] ≥30 or <30 kg/m ^2^ ), diabetes, heart failure, prior SSE, and CHA _2_ DS _2_ VASc score (0–1, ≥2, 2–3, ≥4) were performed. Propensity score weighting was rerun for each sensitivity and subgroup analysis using the same variables as the main analysis. Only the primary effectiveness (SSE) and safety (bleeding-related hospitalizations) outcomes were assessed.

All database management and statistical analysis were performed using SAS version 9.4 (SAS Institute, Cary, NC, United States) and IBM SPSS version 28.0 (IBM Corp., Armonk, NY, United States). A *p* -value <0.05 was considered statistically significant unless otherwise noted. *p* Values for interaction were calculated to test for the presence of statistical interactions. To reduce the chances of obtaining false-positive results (Type I error) because of multiple hypothesis testing, we utilized a Bonferroni corrected *p* -value <0.007 to indicate a statistically significant interaction for subgroup analyses.

### Research Reporting

This report was written in accordance with the reporting of studies conducted using observational routinely collected health data statements for pharmacoepidemiology guidance. 25

## Results

**Fig. 1:**
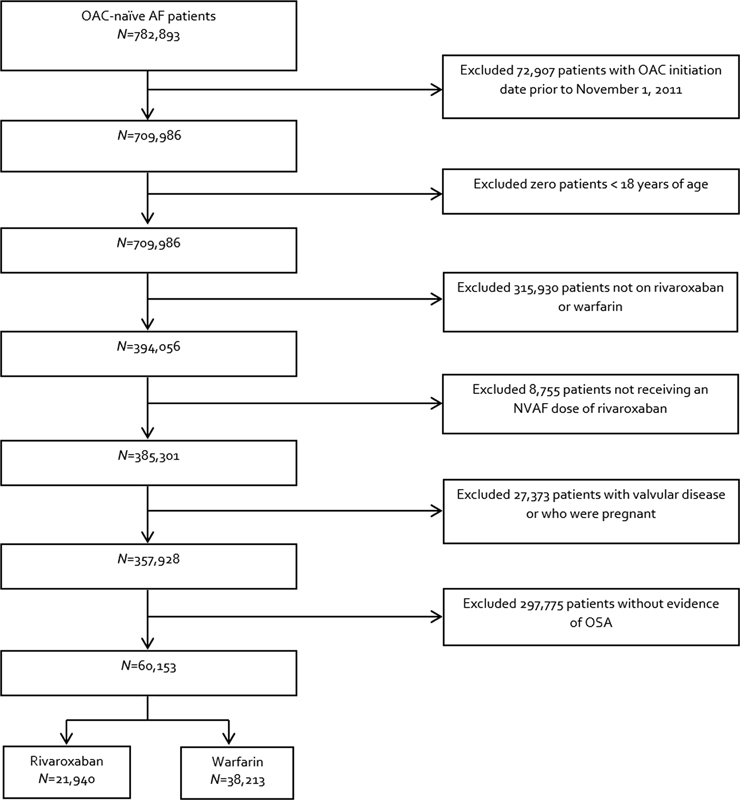
Patient inclusion and exclusion. AF, atrial fibrillation; NVAF, nonvalvular atrial fibrillation; OAC, oral anticoagulant; OSA, obstructive sleep apnea.

We identified 357,928 NVAF patients treated with rivaroxaban or warfarin ( Fig. 1 ). Of these, 60,153 patients (16.8%) were found to have concomitant OSA. This included 21,940 rivaroxaban and 38,213 warfarin-treated patients. Unweighted and weighted baseline characteristics of included patients are depicted in Table 1 .

**Table 1:** Characteristics of included patients in the propensity score overlap weighted analysis

	Unweighted			Propensity score-overlap weighted		
	Rivaroxaban *N* = 21,940	Warfarin *N* = 38,213	ASD	Rivaroxaban *N* = 21,940	Warfarin *N* = 38,213	ASD
Demographics						
Age, years (mean ± SD) a	65.5 ± 10.8	69.2 ± 10.3	–	67.2 ± 10.7	67.6 ± 10.6	–
Age 65–74 y, %	33.9	34.7	0.02	35.2	35.2	0
Age ≥75 y, %	22.0	35.0	0.29	27.6	27.6	0
Female sex, %	30.4	33.3	0.06	32.1	32.1	0
White race, %	88.9	88.9	0	89.2	89.2	0
Hospital frailty score, intermediate risk, %	36.0	41.9	0.12	39.3	39.3	0
Hospital frailty score, high risk, %	12.2	23.7	0.30	15.8	15.8	0
No. Hospitalizations in prior 12 mo (mean ± SD)	0.72 ± 1.35	1.10 ± 1.62	0.26	0.86 ± 1.51	0.86 ± 1.36	0
Continuous or bilevel positive airway pressure in prior 12 mo, %	0.3	0.3	0	0.3	0.3	0
Sleep study in prior 12 mo, %	9.0	7.6	0.05	8.5	8.5	0
Surgical treatment of OSA in prior 12 mo, %	0.3	0.6	0.04	0.3	0.3	0
CHA _2_ DS _2_ VASc score (mean ± SD) a b	2.99 ± 1.63	3.80 ± 1.61	–	3.33 ± 1.66	3.33 ± 1.56	–
CHADS _2_ score (mean ± SD) a c	2.14 ± 1.39	2.81 ± 1.44	–	2.41 ± 1.40	2.41 ± 1.37	–
Modified HASBLED score (mean ± SD) a d	1.99 ± 0.89	2.24 ± 0.88	–	2.09 ± 0.87	2.08 ± 0.86	–
Medical history						
Ablation, %	2.6	3.7	0.06	3.0	3.0	0
Acute coronary syndrome, %	8.0	12.1	0.14	9.5	9.5	0
Acute kidney injury, %	12.9	25.6	0.33	16.8	16.8	0
Anemia, %	18.9	37.7	0.43	24.7	24.7	0
Anxiety, %	18.0	18.1	0.003	18.1	18.1	0
Any bleeding in prior 90 d, %	2.5	5.1	0.14	3.3	3.3	0
Aortic plaque, %	2.7	4.0	0.07	3.0	3.0	0
Asthma, %	13.9	14.9	0.03	14.6	14.6	0
Body mass index 30–39.9 kg/m ^2^ , %	47.0	44.8	0.04	45.6	45.6	0
Body mass index ≥40 kg/m ^2^ , %	30.9	30.2	0.02	31.5	31.5	0
Cardioversion, %	7.8	9.4	0.06	8.1	8.1	0
Carotid stenosis, %	3.2	3.7	0.03	3.7	3.7	0
Carotid endarterectomy and/or stent, %	0.7	1.0	0.03	0.9	0.9	0
Central venous catheter, %	3.5	10.7	0.28	5.2	5.2	0
Chronic obstructive pulmonary disease, %	25.9	35.1	0.20	29.7	29.7	0
Coagulopathy, %	6.1	12.0	0.18	8.0	8.0	0
Coronary artery bypass grafting, %	7.6	15.4	0.25	10.2	10.2	0
Crohn's disease or ulcerative colitis, %	0.8	1.0	0.02	0.9	0.9	0
Chronic venous insufficiency, %	5.3	8.5	0.13	6.4	6.4	0
Dementia, %	2.8	5.2	0.12	3.7	3.7	0
Vascular dementia, %	0.3	0.5	0.03	0.4	0.4	0
Depression, %	19.8	23.1	0.08	21.3	21.3	0
Diabetes mellitus, %	40.3	51.7	0.23	44.7	44.7	0
Diverticular disease, %	7.3	8.2	0.03	7.7	7.7	0
eGFR 30–50 mL/min, %	6.3	11.5	0.18	8.2	8.2	0
eGFR <30 mL/min, %	2.2	11.3	0.37	3.5	3.5	0
Excessive alcohol consumption, %	0.7	1.0	0.03	0.8	0.8	0
Gastroesophageal reflux disease, %	27.8	31.3	0.08	29.3	29.3	0
Gastrointestinal bleed, %	0.6	1.4	0.08	0.8	0.8	0
Heart failure, %	32.5	50.5	0.37	39.7	39.7	0
Headache, %	3.4	2.6	0.05	3.1	3.1	0
Helicobacter pylori, %	0.2	0.3	0.02	0.3	0.3	0
Hemorrhoids, %	4.1	4.3	0.01	4.2	4.2	0
Hypercoagulable state, %	0.9	1.5	0.06	1.1	1.1	0
Hyperlipidemia, %	68.4	73.6	0.11	70.6	70.6	0
Hypertension, %	84.7	89.4	0.14	86.6	86.6	0
Hypothyroidism, %	16.2	20.6	0.11	18.0	18.0	0
Intracranial hemorrhage, %	0.1	0.3	0.05	0.1	0.1	0
Ischemic heart disease, %	38.6	52.8	0.29	44.3	44.3	0
Ischemic stroke, %	5.5	8.0	0.10	6.6	6.6	0
Recent ischemic stroke, %	1.2	1.9	0.06	1.5	1.5	0
Knee or hip surgery, %	0.8	1.5	0.07	1.0	1.0	0
Liver dysfunction, %	3.2	5.3	0.10	3.9	3.9	0
Lower extremity paralysis, %	0.3	0.4	0.02	0.4	0.4	0
Lymphoma, %	1.0	1.2	0.02	1.1	1.1	0
Major adverse limb event, %	6.1	11.1	0.18	7.7	7.7	0
Major amputation, %	0.1	0.4	0.06	0.2	0.2	0
Major bleed, %	0.9	2.6	0.13	1.4	1.4	0
Metastatic cancer, %	1.4	1.8	0.03	1.6	1.6	0
Myocardial infarction, %	12.4	18.3	0.16	14.6	14.6	0
Osteo- or rheumatoid arthritis, %	25.7	27.1	0.03	26.8	26.8	0
Osteoporosis, %	5.3	7.8	0.10	6.4	6.4	0
Percutaneous coronary intervention, %	10.9	15.2	0.13	12.5	12.5	0
Peripheral vascular disease, %	10.9	17.6	0.19	13.1	13.1	0
Pneumonia, %	10.4	16.7	0.57	12.7	12.7	0
Psychosis, %	1.3	2.3	0.08	1.6	1.6	0
Proteinuria, %	2.1	3.1	0.06	2.4	2.4	0
Renal dialysis or transplant, %	0.5	6.6	0.33	1.0	1.0	0
Smoker, %	14.2	13.2	0.03	13.9	13.9	0
Solid tumor, %	9.5	11.9	0.08	10.7	10.7	0
Stroke or systemic embolism, %	6.2	9.9	0.14	7.7	7.7	0
Transient ischemic attack, %	3.3	3.7	0.20	3.5	3.5	0
Varicose veins, %	2.7	3.6	0.05	3.2	3.2	0
Vascular disease (prior MI, PAD or aortic plaque), %	20.7	30.7	0.23	24.3	24.3	0
Medications e						
Amiodarone, %	10.9	17.5	0.19	13.3	13.3	0
ACE inhibitor or ARB, %	61.6	61.2	0.01	62.3	62.3	0
Alpha blocker, %	14.8	18.0	0.09	16.0	16.0	0
Aspirin, %	25.8	29.0	0.07	27.5	27.5	0
Barbiturate, %	1.4	1.4	0	1.4	1.4	0
Benzodiazepine, %	16.9	19.0	0.05	17.8	17.8	0
Beta blocker, %	71.9	74.4	0.06	72.8	72.8	0
Dihydropyridine calcium channel blocker, %	4.4	4.1	0.01	4.2	4.2	0
Digoxin, %	7.8	13.4	0.18	10.1	10.1	0
Diltiazem, %	21.9	19.5	0.06	21.2	21.2	0
Dipeptidyl peptidase-4 inhibitor, %	4.7	4.7	0	4.8	4.8	0
Dronedarone, %	2.5	1.5	0.07	2.1	2.1	0
Estrogen, %	1.8	1,4	0.03	1.5	1.5	0
Glucagon-like peptide-1 analog, %	3.6	2.2	0.08	3.0	3.0	0
Histamine-2 receptor antagonist, %	9.0	11.2	0.07	9.9	9.9	0
Insulin, %	11.7	20.9	0.25	14.6	14.6	0
Levothyroxine, %	14.8	18.3	0.09	16.3	16.3	0
Loop diuretic, %	36.4	55.7	0.39	44.7	44.7	0
Metformin, %	23.3	21.3	0.05	23.5	23.5	0
Nonsteroidal antiinflammatory drug, %	28.5	21.0	0.17	25.6	25.6	0
Other antiarrhythmic agent, %	13.9	8.3	0.18	11.5	11.5	0
Other antidepressant, %	10.0	12.0	0.06	10.9	10.9	0
Other antiplatelet agent, %	0.7	0.9	0.02	0.7	0.7	0
Other cholesterol medication, %	10.4	11.8	0.04	11.0	11.0	0
P2Y12 inhibitor, %	5.2	6.3	0.04	5.8	5.8	0
Proton pump inhibitor, %	36.1	41.4	0.11	37.9	37.9	0
Sodium-glucose cotransporter-2 inhibitor, %	2.1	0.9	0.1	1.6	1.6	0
SSRI or SNRI, %	25.1	26.9	0.04	25.9	25.9	0
Statin, %	57.8	64.2	0.13	60.8	60.8	0
Sulfonylurea or glinide, %	10.1	13.9	0.12	11.8	11.8	0
Thiazide diuretic, %	29.1	26.5	0.06	28.2	28.2	0
Thiazolidinediones, %	1.9	1.9	0	1.9	1.9	0
Verapamil, %	2.1	2.2	0.01	2.2	2.2	0
Warfarin inducer, %	0.9	1.7	0.07	1.2	1.2	0
Warfarin inhibitor, %	5.1	5.1	0	5.2	5.2	0
Time in therapeutic INR range (mean ± SD) a	NA	44.5 ± 28.0	–	NA	47.3 ± 28.3	–

Abbreviations: ASD, absolute standardized difference; INR, international normalized ratio; NA, not available; SD standard deviation; SNRI, serotonin and norepinephrine reuptake inhibitors; SSRI, selective serotonin reuptake inhibitors; ACE, angiotensin-converting enzyme; ARB, angiotensin receptor blockers; eGFR, estimated glomerular filtration rate; MI, myocardial infarction; PAD, peripheral artery disease.

a Covariate not included in the propensity score model.

b CHA _2_ DS _2_ VASc = congestive heart failure, 1 point; hypertension, 1 point; age ≥ 75 years, 2 points; diabetes mellitus, 1 point; previous stroke, transient ischemic attack, or thrombo-embolism, 2 points; vascular disease, 1 point; age 65–74 years, 1 point; female sex, 1 point.

c CHADS _2_ = congestive heart failure, 1 point; hypertension, 1 point; age ≥ 75 years, 1 point; diabetes mellitus, 1 point; previous stroke or transient ischemic attack, 2 points.

d Modified HASBLED = hypertension, 1 point; age > 65 years, 1 point; stroke history, 1 point; bleeding history or predisposition, 1 point; labile international normalized ratio, not assessed; ethanol or drug abuse, 1 point; drug predisposing to bleeding, 1 point.

e Either prescribed or self-reported by patients.

After propensity score overlap weighting, the rivaroxaban and warfarin cohorts were identical (ASD = 0 for all). The mean age of patients included in the study was approximately 67 years. Only 0.3% of patients were receiving treatment with continuous or bilevel positive airway pressure, and 0.3% underwent a surgical procedure to treat OSA during the prior 12 months. Aspirin was used concomitantly with anticoagulation in 27.5% of patients, while 6.5% of patients utilized a P2Y12 inhibitor or another antiplatelet agent. The mean CHA _2_ DS _2_ VASc score was 3.33, and mean modified HASBLED score was 2.09 for rivaroxaban and 2.08 for warfarin patients. The 15 mg dose was used in 20.1% of rivaroxaban patients. Warfarin patients spent an average of 47.3 ± 28.3% of their time in the target therapeutic international normalized ratio range (using linear interpolation and assuming a target range of 2.0–3.0).

**Fig. 2:**
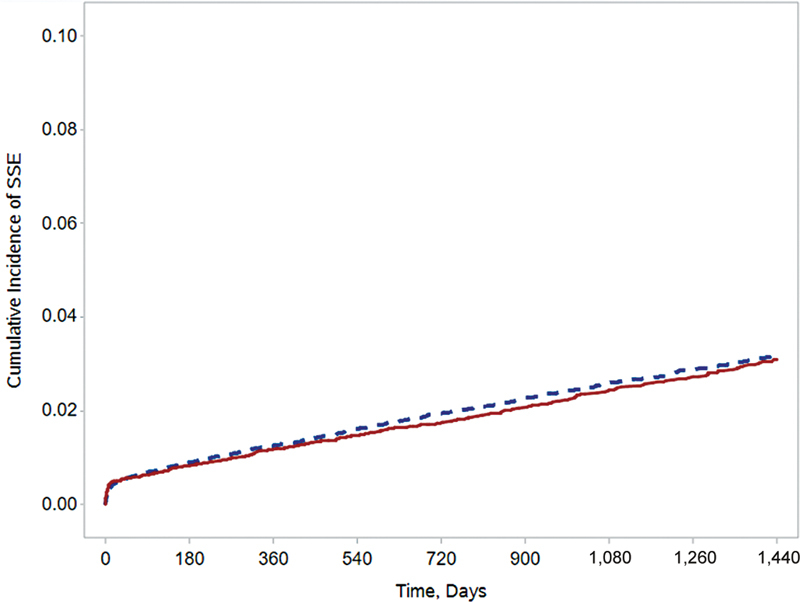
Kaplan–Meier curve for stroke or systemic embolism. Red/solid line represents rivaroxaban; blue/dashed line represents warfarin.

**Fig. 3:**
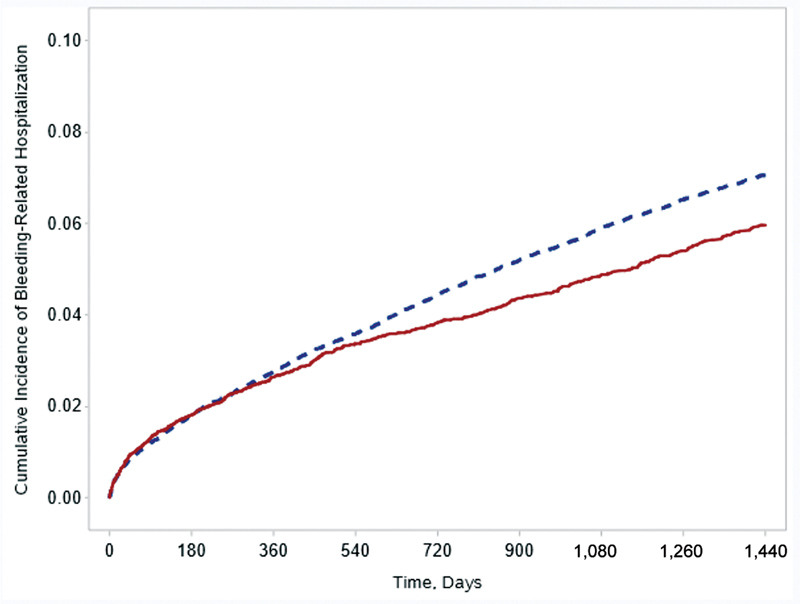
Kaplan–Meier curve for bleeding-related hospitalization. red/solid line represents rivaroxaban; blue/dashed line represents warfarin.

Mean follow-up was 1,273 ± 837 days for the entire study cohort and similar between the rivaroxaban (1,290 ± 814 days) and warfarin (1,255 ± 859) groups (ASD = 0.04). Propensity score-overlap weighted proportional hazards regression did not show a significant difference in the primary effectiveness outcome of SSE between rivaroxaban and warfarin (0.74 vs. 0.81%/y, HR = 0.92, 95%CI = 0.82–1.03; Table 2 and Fig. 2 ). The similar rate of ischemic stroke alone was observed between groups (HR = 1.01 95% CI = 0.88–1.16). For the primary safety outcome of bleeding-related hospitalization, rivaroxaban was associated with a decreased rate (1.52 vs. 1.81%/y; HR = 0.85, 95% CI = 0.78–0.92; Fig. 3 ). Both intracranial (HR = 0.76, 95% CI = 0.62-0.94) and extracranial bleeding (HR = 0.89, 95% CI =  0.62–0.84) were reduced with rivaroxaban versus warfarin use.

**Table 2:** Effectiveness and safety outcomes

	Rivaroxaban *N* = 21,940 (%/y)	Warfarin *N* = 38,213 (%/y)	OLW-HR 95%CI
Primary outcomes			
Stroke or systemic embolism	0.74	0.81	0.92 (0.82–1.03)
Bleeding-related hospitalization	1.52	1.81	0.85 (0.78–0.92)
Secondary outcomes			
Ischemic stroke	0.52	0.51	1.01 (0.88–1.16)
Intracranial hemorrhage	0.22	0.28	0.76 (0.62–0.94)
Extracranial bleed	1.28	1.44	0.89 (0.81–0.97)

Abbreviations: CI, confidence interval; HR, hazard ratio; *N* , sample size; OLW, overlap weighting.

Upon sensitivity analysis, utilization of sIPTW instead of OLW did not impact the results for the SSE or bleeding-related hospitalization outcomes ( Table 3 ). When restricting the population to men with a CHA _2_ DS _2_ VASc score ≥2 or women with a score ≥3, based on treatment recommendations from the 2019 AHA guidelines, 1 rivaroxaban was associated with a significant 33% reduction in the risk of SSE, as well as a more profound reduction in bleeding-related hospitalization (HR = 0.57, 95% CI = 0.53–0.62) compared to the full population analysis. Similar findings were observed upon subgroup analysis comparing CHA _2_ DS _2_ VASc score of 0 to 1 versus ≥2 (regardless of sex). Patients with a CHA _2_ DS _2_ VASc score ≥2 receiving rivaroxaban compared to warfarin were less likely to develop SSE (HR = 0.65, 95% CI = 0.59–0.73; p-interaction = 0.10 vs. CHA _2_ DS _2_ VASc score of 0–1). Rivaroxaban users with a CHA _2_ DS _2_ VASc score ≥2 were also significantly less likely to experience a bleeding-related hospitalization (HR = 0.56, 95% CI = 0.52–0.60), and the p-interaction value of 0.002, suggests there was a presence of a statistical interaction suggesting rivaroxaban is associated with less bleeding-related hospitalizations in patients with CHA _2_ DS _2_ VASc scores ≥2 versus 0 to 1. Other subgroup analyses did not find a statistically significant interaction across any subgroup for either the SSE or bleeding-related hospitalization outcome.

**Table 3:** Results of sensitivity and subgroup analyses

	Stroke or systemic embolism				Bleeding-related hospitalization			
Sensitivity or subgroup analysis	Rivaroxaban %/y	Warfarin %/y	HR (95%CI)	Heterogeneity *p* -value a	Rivaroxaban %/y	Warfarin %/y	HR (95%CI)	Heterogeneity *p* -value ^a^
Overall, *N* = 60,153	0.74	0.81	0.92 (0.82–1.03)	–	1.52	1.81	0.85 (0.78–0.92)	**–**
Sensitivity analyses								
sIPTW, *N* = 60,153	0.84	0.93	0.90 (0.78–1.03)	–	1.79	2.13	0.84 (0.76–0.94)	**–**
Moderate-to-High CHA _2_ DS _2_ VASc, *N* = 51,346	0.79	1.15	0.67 (0.60–0.74)	–	1.55	2.63	0.57 (0.53–0.62)	**–**
Subgroup analyses								
Age				0.23				0.05
≥75 y, *N* = 18,198	1.21	1.25	0.98 (0.81–1.18)		2.51	2.64	0.96 (0.84–1.01)	
< 75 y, *N* = 41,995	0.58	0.69	0.85 (0.73–0.98)		1.20	1.59	0.76 (0.68–0.84)	
Sex				0.77				>0.99
Female, *N* = 19,402	0.81	0.91	0.90 (0.74–1.10)		1.63	1.93	0.85 (0.74–0.98)	
Male, *N* = 40,751	0.71	0.78	0.93 (0.81–1.08)		1.47	1.74	0.85 (0.77–0.94)	
Body mass index				0.66				0.46
< 30 kg/m ^2^ , *N* = 14,370	0.96	1.01	0.96 (0.77–1.20)		1.75	1.98	0.89 (0.76–1.05)	
≥30 kg/m ^2^ , *N* = 45,783	0.68	0.75	0.91 (0.79–1.04)		1.46	1.76	0.83 (0.76–0.91)	
Diabetes				0.23				0.05
Yes, *N* = 28,599	0.83	0.97	0.86 (0.73–1.01)		1.73	2.23	0.79 (0.70–0.88)	
No, *N* = 31,554	0.69	0.69	0.99 (0.84–1.16)		1.38	1.49	0.93 (0.83–1.04)	
Heart failure				0.21				0.09
Yes, *N* = 26, 415	1.01	102	0.99 (0.84–1.17)		2.30	2.57	0.91 (0.81–1.01)	
No, *N* = 33,738	0.60	0.70	0.86 (0.73–1.00)		1.10	1.41	0.79 (0.70–0.89)	
Prior stroke or systemic embolism				0.06				0.23
Yes, *N* = 5,154	3.66	3.40	1.10 (0.89–1.35)		2.65	2.73	0.97 (0.76–1.24)	
No, *N* = 54,999	0.54	0.63	0.86 (0.75–0.99)		1.44	1.74	0.83 (0.76–0.91)	
CHA _2_ DS _2_ VASc b (low vs. moderate-high)				0.10				0.002
0–1, *N* = 6,948	0.23	0.22	1.06 (0.60–1.87)		0.60	0.59	1.03 (0.71–1.49)	
≥2, *N* = 53,205	0.75	1.11	0.65 (0.59–0.73)		1.48	2.57	0.56 (0.52–0.60)	
CHA _2_ DS _2_ VASc b (low vs. moderate vs. high)								
0–1, *N* = 6,948	0.23	0.22	1.06 (0.60–1.87)	0.24	0.60	0.59	1.03 (0.71–1.49)	0.01
2–3, *N* = 23, 674	0.44	0.56	0.79 (0.64–0.98)		1.06	1.48	0.72 (0.62–0.83)	
≥4, *N* = 29,531	1.29	1.33	0.98 (0.85–1.13)		2.40	2.65	0.92 (0.83–1.01)	

Abbrevaitions: CI, confidence interval; HR, hazard ratio; *N* , sample size; sIPTW, stabilized inverse probability of treatment weighting.

a Due to multiple hypothesis testing, a *p* -value for heterogeneity of <0.007 was required to demonstrate a statistically significant interaction in the subgroup analyses.

b CHA _2_ DS _2_ VASc = congestive heart failure, 1 point; hypertension, 1 point; age ≥ 75 years, 2 points; diabetes mellitus, 1 point; previous stroke, transient ischemic attack, or thrombo-embolism, 2 points; vascular disease, 1 point; age 65–74 years, 1 point; female sex, 1 point.

## Discussion

Our study utilized detailed EHR data to evaluate more than 60,000 patients with NVAF and comorbid OSA newly started on OAC with either rivaroxaban or warfarin with a mean follow-up period of approximately 3.5 years. Rivaroxaban was found to have a similar SSE risk compared to warfarin in the analysis of all NVAF patients with OSA; however, sensitivity analysis did show rivaroxaban to be associated with a 33% significant reduction in SSE versus warfarin when the population was restricted to patients with a moderate-to-high risk of SSE based on CHA _2_ DS _2_ VASc (men with a CHA _2_ DS _2_ VASc score ≥2 or women with a score ≥3). Similar findings were seen in the subgroup analysis of patients stratified by CHA _2_ DS _2_ VASc score ≥2 irrespective of sex. Rivaroxaban was associated with a significant 15% relative hazard reduction in any bleeding-related hospitalizations compared to warfarin. This outcome was particularly driven by a 24% and 11% hazard reduction in intracranial and extracranial events, respectively. Outcomes did not differ when sIPTW was utilized instead of OLW or across non-CHA _2_ DS _2_ VASc score stratified subgroups evaluated.

To our knowledge, this is the first study to evaluate the comparative effectiveness and safety of a DOAC versus a VKA in NVAF patients with concomitant OSA. The results of our present real-world analysis are generally consistent with the overall results of the 14,000+ patient Rivaroxaban Once Daily Oral Direct Factor Xa Inhibition Compared with Vitamin K Antagonism for Prevention of Stroke and Embolism Trial in Atrial Fibrillation (ROCKET-AF). 26 In both ROCKET-AF and the present real-world study, the rates of SSE were shown to be similar between rivaroxaban and warfarin-treated patients, with reductions in the risk of ICH in patients receiving rivaroxaban (33% risk hazard reduction in ROCKET-AF and a 24% relative hazard reduction in the present study). Our finding of a significant 15% reduction in any bleeding-related hospitalization was not anticipated based on the results of ROCKET-AF, which showed no difference in major bleeding. This difference may be explained, at least in part, by the decreased amount of time in therapeutic range (TTR) in warfarin users in our study (47.3 ± 28.3%), which was approximately 8% lower than the mean TTR seen in ROCKET-AF (approximately 55%). 26 Of note, prior data suggest that OSA patients are more difficult to maintain in therapeutic range, while on a VKA. 27

Whether a diagnosis of concomitant OSA independently increases the risk of ischemic stroke in patients with NVAF remains unclear, with conflicting evidence being published. 8 10 11 Yaranov and colleagues assessed over 300 patients with AF as part of a chart review and identified a significant 3.65-fold increased risk of stroke in patients with OSA compared to those without. 8 The overall impact a diagnosis of OSA on outcomes in NVAF patients was assessed in an analysis of the Outcomes Registry for Better Informed Treatment of Atrial Fibrillation (ORBIT-AF). 10 Patients with OSA and AF in ORBIT-AF were younger (69 vs. 76 years; *p* < 0.0001) and had a more extensive past medical history, including an increased frequency of history of diabetes and obesity than those without OSA. This finding was consistent with the OSA population in our analysis who had a similar age (mean approximately 67 years) with elevated percentages of patients with obesity or BMI ≥ 30 (77.1%), and diabetes (44.7%), all of which are independent risk factors for increased rates of cardiovascular outcomes in NVAF. 28 29 Despite these differences in baseline characteristics, the adjusted risk of major adverse cardiovascular events (composite of cardiovascular death, MI, stroke/transient ischemic attack) in ORBIT-AF were found to be similar in those with and without OSA (HR = 1.07, 95% CI = 0.85–1.34). 10 An additional retrospective analysis of the Danish national registry by Koch et al 11 assessed the risk of ischemic stroke over a 5-year period in patients with first-time AF (1,766 had prior sleep apnea and these were matched with 7064 without sleep apnea) and found that a history of sleep apnea was not associated with an increased risk of ischemic stroke (HR = 1.06, 95% CI = 0.86–1.30). Interestingly, our study population of NVAF patients, all with OSA, was associated with low rates of ischemic stroke in both groups (0.52%/y in the rivaroxaban and 0.51%/y in the warfarin cohort). This may be attributed to patients' relatively younger age and low CHADS _2_ /CHA _2_ DS _2_ VASc scores, 1 as well as the fact that Optum's EHR repository does not encompass all institutions and therefore may have missed relevant follow-up events. 13 Rates of ischemic stroke did increase (as high as 1.15%/y in the warfarin cohort) when the population was restricted to men with a CHA _2_ DS _2_ VASc score ≥2 or women with a score ≥3.

There are no formal recommendations on the choice of anticoagulant in AF patients with concomitant OSA, though both U.S. and European guidelines recommend that patients who are eligible for OAC receive a DOAC in preference to a VKA, except in patients with mechanical heart valves or moderate-to-severe mitral stenosis (class 1A recommendations). 1 30 Both U.S. and European guidelines 1 30 recommend OSA be screened for in AF patients and properly managed when found to reduce symptoms of AF. Continuous PAP is considered the therapy of choice for OSA, with observational studies and meta-analyses suggesting appropriate PAP treatment of OSA may improve rhythm control in AF patients. 30 Interestingly, our study identified very low rates of PAP use across our OSA population. This finding may be explained through several mechanisms. First, studies have suggested that PAP therapy is underprescribed in OSA. 31 Moreover, inconvenience and other device factors, lack of perceived benefit, poor disease perception, embarrassment, and cost/insurance coverage have been shown to be major barriers to the availability and adherence/persistence to PAP treatment. 32 Only 8.5% of patients in our study underwent a sleep study in the prior 12 months. This may suggest that many patients included in our study had OSA diagnosed one or more years ago. Consequently, patients trying PAP therapy at some point after their OSA diagnosis but who were non-persistent, would not have been classified as being on PAP therapy due to our limited look-back period to identify baseline characteristics and treatments. Finally, it is also possible that oral appliances were used as an alternative to PAP therapy. Though not effective for severe OSA (apnea-hypopnea index ≥ 30 events/h), oral appliances are indicated for patients with mild-to-moderate OSA who either prefer them over PAP therapy or who have failed or rejected PAP therapy. 33 Unfortunately, we were not able to assess OSA severity in the present study.

There are several limitations of this study worth discussing. The study's nonrandomized and retrospective study design may result in misclassification and confounding bias. Strategies for minimizing the probability of misclassification implemented in our study included using validated coding schema and leveraging EHR laboratory and clinical observation data. Billing codes for OSA are accurate at identifying patients with OSA, but less helpful at ruling out those without disease. 14 15 Consequently, our analysis likely underestimated the proportion of NVAF patients with OSA (and excluded some cases from the analysis). This may be further exacerbated by the fact that OSA has been historically underdiagnosed. 31 32 The data set used in our study also did not allow for the determination of duration or severity of the OSA. Propensity score-overlap weighting was utilized to reduce the risk of confounding bias, 21 and while the ASD was zero for all covariates after weighting, residual confounding on covariates not collected and entered in the model cannot be ruled out. Next, the observational nature of this study prohibited any control over warfarin dosing. As previously noted, the TTR for warfarin patients in our study was lower (approximately 47%) than typically seen in RCTs and in dedicated anticoagulation clinics, 26 34 though this is a common finding of studies assessing TTR in routine clinical practice and may be further exacerbated by the difficulty of maintain goal TTR in OSA patients. 27 The results of this study should be viewed as being most generalizable to a U.S. population, as the EHR data was limited to the United States and practice patterns for the treatment of AF and OSA may vary from country to country. 13 Furthermore, the extent to which these results are applicable to those patients receiving successful PAP therapy could not be determined with this dataset. The data set used also lacked information on prescription medication claims and instead only provide data on medications prescribed or self-reported. 13 While the latter is beneficial in identifying important medications that may otherwise be over the counter and not identified in claims data (e.g., aspirin), the lack of prescription claims makes an accurate assessment of anticoagulant adherence challenging. Therefore, analyses in the present study utilize an intent-to-treat methodology only. Lastly, Optum's EHR repository solicits data from both insured and uninsured patients, but it does not encompass all institutions; therefore, relevant follow-up events could potentially be missed. 13

## Conclusion

In patients with NVAF and concomitant OSA, rivaroxaban-treated patients had similar SSE compared to warfarin but was associated with reductions in any, intracranial, and extracranial bleeding-related hospitalizations. When the population was restricted to only patients with a moderate-to-high risk of SSE, rivaroxaban was associated with significant reductions in both SSE and bleeding-related hospitalization. These data should provide prescribers with additional confidence in selecting rivaroxaban in NVAF patients who have OSA at the time of anticoagulation initiation.
